# Arabinogalactan Proteins Are Involved in Salt-Adaptation and Vesicle Trafficking in Tobacco by-2 Cell Cultures

**DOI:** 10.3389/fpls.2017.01092

**Published:** 2017-06-20

**Authors:** Enrique Olmos, Jesús García De La Garma, Maria C. Gomez-Jimenez, Nieves Fernandez-Garcia

**Affiliations:** ^1^Department of Abiotic Stress and Plant Pathology, Centro de Edafología y Biología Aplicada del Segura (CSIC)Murcia, Spain; ^2^Department of Plant Physiology, Faculty of Science, University of ExtremaduraBadajoz, Spain

**Keywords:** arabinogalactan proteins, yariv reagent, salinity adaptation, phospholipase C, vesicle trafficking

## Abstract

Arabinogalactan proteins (AGPs) are a highly diverse family of glycoproteins that are commonly found in most plant species. However, little is known about the physiological and molecular mechanisms of their function. AGPs are involved in different biological processes such as cell differentiation, cell expansion, tissue development and somatic embryogenesis. AGPs are also involved in abiotic stress response such as salinity modulating cell wall expansion. In this study, we describe how salt-adaptation in tobacco BY-2 cell cultures induces important changes in arabinogalactan proteins distribution and contents. Using the immuno-dot blot technique with different anti-AGP antibodies (JIM13, JIM15, and others), we observed that AGPs were highly accumulated in the culture medium of salt-adapted tobacco cells, probably due to the action of phospholipases. We located these AGP epitopes using immunogold labeling in the cytoplasm associated to the endoplasmic reticulum, the golgi apparatus, and vesicles, plasma membrane and tonoplast. Our results show that salt-adaptation induced a significant reduction of the cytoplasm, plasma membrane and tonoplast content of these epitopes. Yariv reagent was added to the control and salt-adapted tobacco cell cultures, leading to cell death induction in control cells but not in salt-adapted cells. Ultrastructural and immunogold labeling revealed that cell death induced by Yariv reagent in control cells was due to the interaction of Yariv reagent with the AGPs linked to the plasma membranes. Finally, we propose a new function of AGPs as a possible sodium carrier through the mechanism of vesicle trafficking from the apoplast to the vacuoles in salt-adapted tobacco BY-2 cells. This mechanism may contribute to sodium homeostasis during salt-adaptation to high saline concentrations.

## Introduction

Salt stress is one of the major factors that affect plant development and production. Salinization of agricultural lands has increased progressively in recent decades. Glycophyte crops lose their vigor even in slightly saline soils and most crops are negatively affected by moderate salinity (Munns and Gilliham, 2015). Plant growth and development are seriously affected by salinity. Plants develop various physiological and biochemical mechanisms in order to avoid the negative effect of salinity. In recent decades biologist have focused their studies on the mechanisms that regulate salt tolerance, such as ion homeostasis and compartmentalization, ion uptake and distribution, biosynthesis of osmoprotectants, antioxidant enzymes, hormones, etc., (Munns and Tester, 2008). However, little is known about the role of the different components of cell walls during salt adaptation to highly saline conditions. One of the most interesting features of plant growth is the precise and coordinated regulation as the cell size increased. Plant cell growth is mainly regulated by the addition and modification of macromolecules that compose cell walls (Tenhaken, 2015). A primary cell wall is typically composed of cellulose (25–30%), pectins (30%), hemicellulose (20–30%), and proteins (1–10%). Plant cell walls contain many different proteins, and among them, proteoglycans and glycoproteins are abundantly represented. The hydroxyproline-rich glycoprotein (HRGP) superfamily comprises three main families, arabinogalactan proteins (AGPs, highly glycosylated), extensins (moderately glycosylated), and prolin-rich proteins (lightly glycosylated). Chimeric and hybrid forms of HRGPs can also be included in this superfamily (Showalter et al., 2010). AGPs are a complex family of proteoglycans of high molecular weight that are located in the cell walls, plasma membranes, tonoplast, and the intercellular spaces of plants. AGPs are glycoproteins that are to be found ubiquitous in bryophytes and higher plants (Ellis et al., 2010). The AGP family is subdivided into six subfamilies: classical AGPs, AGP peptides, lysine-rich AGPs, fasciclin-like AGPs (FLAs), non-classical AGPs and chimeric AGPs (Showalter et al., 2010). AGPs are composed of a protein backbone (1–10%) and the carbohydrate moiety (90–99%); the most abundant carbohydrates are arabinose and galactose residues, although some other carbohydrates, such as rhamnose, fucose, xylose, and glucuronic acid, are also present. Most of the hydroxyproline residues are O-glycosylated by arabino-3,6-galactans, but short arabinosides have also been found.

AGPs are anchored to the plasma membrane through a C-terminal glycosylphosphatidylinositol (PI) lipid. AGPs can be cleaved by the action of phospholipases C and can then be displaced to the periplasm, cell walls, and intercellular spaces. Lamports et al. proposed that AGPs may be acting in the cell wall matrix as pectin plasticizers, so affecting cell wall expansion (Lamport et al., 2006). AGPs have also been detected in numerous biological processes such as cell differentiation, tissue development, somatic embryogenesis and calcium capacitors (Lamport and Varnai, 2013).

Different tools have been developed to study AGPs. Yariv reagent is the most frequently compound used for the purification of AGPs (Lamport et al., 2006). AGPs have the ability to specifically bind the Yariv reagent but the mechanism is not totally understood. On the other hand, the development of a huge number of specific monoclonal antibodies directed against specific epitopes of AGPs constitutes the main tool for sub-cellular location of proteoglycans (Samaj et al., 2000). Moreover, these antibodies have been successfully used for AGPs semi-quantification by immuno-dot blot (Willats et al., 1998).

Plant cell cultures such as tobacco BY-2 have been used as cellular models to study the physiological and molecular mechanisms that regulate salt-adaptation to high saline concentrations (Garcia de la Garma et al., 2015). Lamport et al. (2006) using tobacco cell cultures adapted to high saline concentrations, reported a decrease in plasma membrane-bound AGPs, but an increase in AGP accumulation in the culture medium in BY-2 cells adapted to salt stress. These authors used the Yariv reagent to analyze the cellular distribution of total AGPs in BY-2 cells adapted to salt stress through cell fractioning and quantification. In our study, we investigated the involvement of AGPs during salt adaptation in cell cultures of *Nicotitana tabacum* cv. BY2. We have analyzed the different contribution to salt-adaptation of the AGP exocytic and endocytic pathways using several monoclonal antibodies against AGPs, determining subcellular location of AGPs by immunogold labeling and semi-quantification of AGPs in the culture medium by immuno-dot blot. Following these techniques, we have observed that salt adaptation induced a high accumulation of AGPs in the culture medium. We propose the involvement of phospholipase C as a key enzyme, regulating the AGP excretion to the culture medium. We also propose a new role of AGPs as sodium carriers through vesicle trafficking from the plasma membrane to the tonoplast.

## Materials and methods

### Cell culture

BY-2 cells (derived from *Nicotiana tabacum* L. cv. Bright Yellow-2) were grown in a rotary shaker at 130 rpm at 26°C in darkness in a modified Murashige-Skoog medium. The control cells were sub-cultured to fresh medium weekly. Tobacco BY-2 cells were adapted to 258 mM (15 gL^−1^) salt by initial transfer to media containing 86 mM (5 gL^−1^) NaCl for 1 month, 172 mM (10 gL^−1^) NaCl for several weeks and then to 258 mMNaCl-yielding adapted lines cultured for at least 6 months (Garcia de la Garma et al., 2015). The adapted cells were sub-cultured to fresh culture medium at 2 weekly intervals due to a lower growth rate.

### Ultrastructure

For studying cells ultrastructure, the samples were embedded in Spurr resin as described in Garcia de la Garma et al. (2015). Briefly, samples were fixed for 2.5 h at 4°C in a 0.1 M Na-phosphate buffered (pH 7.2) mixture of 2.5% glutaraldehyde and 4% paraformaldehyde. Tissue was post-fixed with 2% osmium tetroxide for 2 h. The samples were then dehydrated in a graded alcohol series and propylene oxide and embedded in Spurr's resin. Blocks were sectioned on a Leica EM UC6 ultramicrotome, collected on formvar-coated copper grids and stained with uranyl acetate followed by lead citrate. Ultra-thin sections were examined using a Philips Tecnai 12 transmission electron microscope.

### Immunogold labeling of AGPs

Samples of control and salt-adapted cells were fixed in 4% paraformaldehyde and 0.25% glutaraldehyde in 0.1 M phosphate buffer (pH 7.2), for 2 h at 4°C, rinsed in the same buffer and dehydrated in an ethanol series. Samples were embedded in LR White as described by Fernandez-Garcia et al. (2009).

Ultrathin sections (70 nm) were obtained with a Leica EM UC6 ultramicrotome (Leica Mikrosysteme, Hernalser Hauptstraße, Vienna, Austria) and collected on formvar-coated nickel grids. The grids were placed in phosphate-buffered saline (PBS) with 5% bovine serum albumin (BSA) for 30 min at room temperature and then incubated for 2 h with the primary monoclonal antibodies (AGPs:LM2, JIM4, JIM13, JIM15; Plant Probes, UK) diluted (1:20) in PBS containing 5% BSA. The sections were washed three times in PBS and incubated with the secondary antibody (goat anti-rat coupled with 15-nm colloidal gold, BioCell International) diluted 1:50 in PBS supplemented with 1% BSA. The grids were washed in buffer and distilled water and dried at 37°C. Ultra-thin sections were stained with uranyl acetate followed by lead citrate. Samples were observed using a Philips Tecnai 12 electron microscopy.

### Quantitative analysis of immunogold labeling

Morphometrical data have been obtained as described by Fernandez-Garcia et al. (2009). Images were directly captured using at CCD SIS MegaView camera and were analyzed using the software AnalySIS® version 3.0. (Soft Imaging System GmbH, Münster, Germany). Gold particles were manually identified and quantified with the software AnalySIS®. The cytoplasm area, plasma membrane and tonoplast length were manually measured using the software AnalySIS®. Vacuole density of labeling was very low, 0.22 gold particles per μm^2^, similar to the unspecific background labeling (0.3 gold particles per μm^2^), and therefore were not statistically evaluated. For statistical analysis at least 10 different cells per treatment were examined. The data were statistically analyzed using the software Statistix 8 (NH Analytical Software, Roseville, MN, USA). Statistical differences were analyzed by the Tukey test (*p* < 0.05).

### Immuno-dot blot assays

Cell culture media were filtered through a 0.22 μm filter (Millex-GP, Millipore). 1 μL of culture medium (each sample was diluted to obtain the same ratio of fresh cell weight/cell culture volume) were dotted onto nitrocellulose (Protan Membrane, Sigma-Aldrich), air dried, blocked with 3% dried skimmed nonfat milk powder (MP) dissolved in PBS (MP/PBS), pH 7.4, for 1 h, washed in PBS, and subsequently incubated during 2 h with the same antibodies used for immunogold labeling diluted 1/10 in MP/PBS. After washing with PBS, sheet was incubated with the secondary antibody (anti-rat IgG conjugated with horseradish peroxidase, Sigma-Aldrich) diluted 1/1,000 in MP/PBS during 1.5 h. Sheet was washed with PBS and after several washings; sheet was incubated with the enzymatic substrate.

### Microarray analysis for AGPs

Four sets of biological replicates were collected independently for control and salt-adapted cells growing at log phase. An Affymetrix Custom Chip designed by Edwards et al. (2010) was used. Total RNA was extracted with QUIAGEN RNAeasy extraction kit according to the supplier's instructions. The Student's *t*-test (*p*-value) was used as a parametric test and the Benjamini-Hochberg False Discovery Rate (FDR > 0.01) procedure was used to control the certainty level using the Partek Express software. Genes with at least 1.5-fold difference in their expression levels were considered to be differentially expressed. Gene annotation was developed using Blast2GO. The raw microarray data and the detailed protocol were deposited in the Gene Expression Omnibus database with GEO ID: GSE42562.

### Confocal laser scanning microscopy and bright field microscopy

Plasma membrane was labeled by incubation of the samples for 2 min with FM4-64 (16 μM). Fluorescence images were obtained with a Leica TCS SP2 AOBS spectral confocal microscope. Samples were excited with the 488 nm line of an argon laser. FM4-64 dye emission was collected at 650 ± 30 nm. The fluorescence was visualized in a single optical section of cells. Labeling of sodium was carried out with Asante Natrium-Green, as described by Garcia de la Garma et al. (2015).

Optical images were obtained with a Leica DMR microscope equipped with DIC system.

### Yariv reagent treatments

Cell cultures were treated with 100 μM of β-D-glucosyl Yariv (Biosupplies, Australia) for 24 h and then washed with fresh culture medium. Samples for ultrastructure, immunogold labeling and confocal laser scanning microscopy were processed as described above. Cell viability was analyzed by staining with Evans Blue (0.025%). Cell viability was calculated as the percentage of live cells. More than 900 cells were counted and three independent experiments were performed.

### Inhibition of phospholipase C

We have analyzed the involvement of phospholipases C in the regulation of AGPs excretion to the culture medium. 100 μM of the aminosteroid U73122 (Sigma-Aldrich), a specific phospholipase C inhibitor, was applied to the control and salt-adapted cells during 24 h. The inactive analog U73343 (Sigma-Aldrich) was also used as a negative control. Inhibitors were dissolved in 500 μM of dimethylsulfoxide (DMSO). Similarly, control and salt-adapted cells were also treated with the same amount of DMSO solvent as a control. Immuno-dot blot assay of the culture medium was developed as described above. At least three independent experiments, each with five replicates, were conducted for each treatment.

## Results

### AGPs excretion to the culture medium

**Figure 1:**
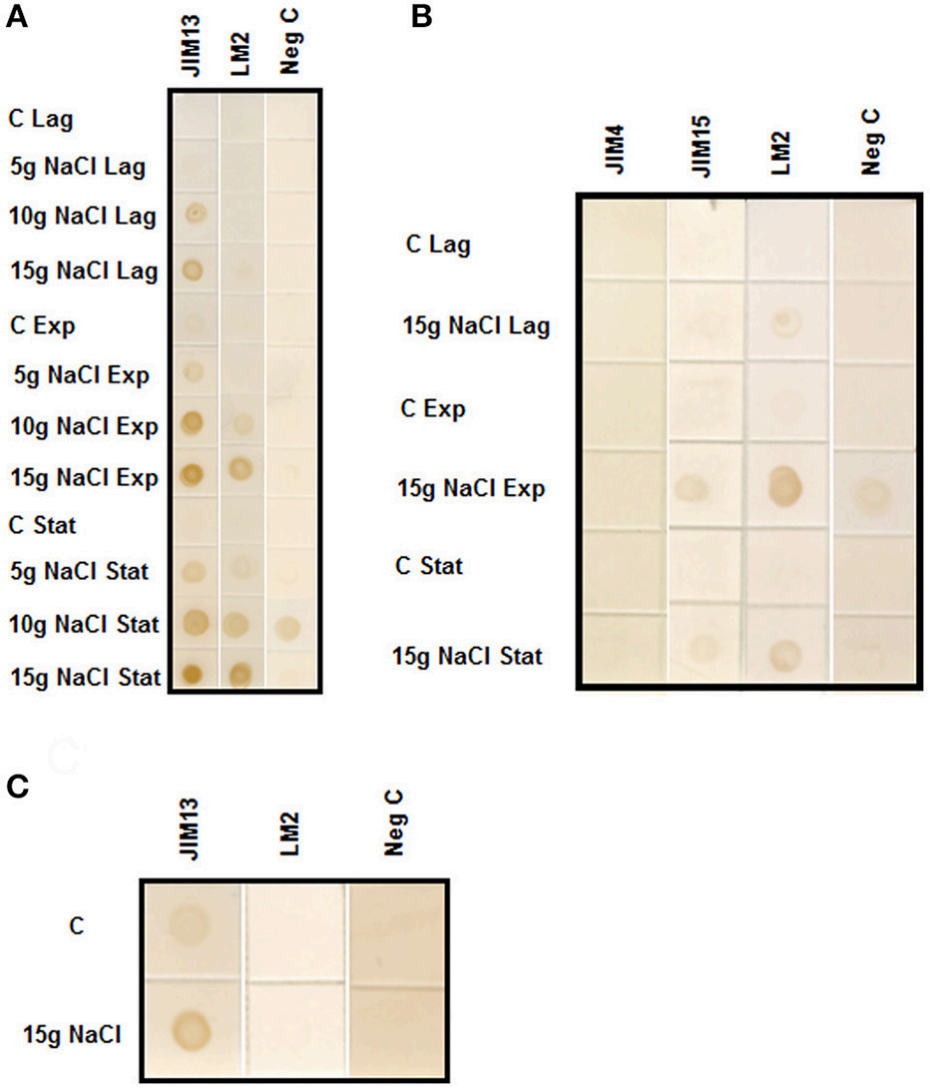
**(A)** Immuno-dot blot analysis with JIM13 and LM2 antibodies on samples prepared from culture medium of control and tobacco BY-2 cells adapted to growth in 0, 5, 10, and 15 gL^−1^ of NaCl. Samples were collected from culture medium of cells growing at Lag, Exponential (Exp) and Stationary (Stat) phases. **(B)** Immuno-dot blot analysis with JIM15, JIM4, and LM2 antibodies on samples prepared from culture medium of control and 15 gL^−1^ salt-adapted tobacco BY-2 cells. Samples were collected from culture medium of cells growing at Lag, Exponential (Exp) and Stationary (Stat) phases. **(C)** Immuno-dot blot analysis of arabinogalactan protein release from control and tobacco BY-2 cells adapted to growth in 15 gL^−1^ of NaCl. The same weight of washed cells from control and adapted cells were cultured in fresh medium. After 24 h, cells were centrifuged and culture media were assayed with JIM13 and LM2 antibodies.

We have used several different monoclonal antibodies against different epitopes of AGPs: LM2 that recognize β-linked-GlcA in the glycan moiety of AGP and JIM4, JIM13, and JIM15 that recognize the AGP glycan. We have followed the different growth phases of the cultures (lag, exponential and stationary) in cells adapted to 5, 10, and 15 gL^−1^ of NaCl. Figure 1A shows that AGPs excretion was proportional to the level of salt-adaptation. Cell adapted to 15 gL^−1^ of NaCl showed the higher accumulation of AGPs during the exponential and stationary phases. Both epitopes were present in the culture media; however, JIM13 showed a higher labeling in all the samples analyzed. We extended our analysis of AGPs using another two monoclonal antibodies, JIM4, and JIM15 that recognize different epitopes of the AGP glycan (Yates et al., 1996). The epitope JIM4 was not present in the culture medium; however, the epitope JIM15 was observed but at a lower labeling than JIM13 and showing a similar pattern to JIM13 and LM2 (Figure 1B).

To analyze the rate of AGPs excretion, the same weight of control and salt-adapted cells growing during the exponential phase were washed with fresh medium and cultured for 24 h. The immuno-dot blot was developed with the antibodies JIM13 and LM2. The results showed that salt-adapted cells had a higher rate of AGPs excretion than control cells (Figure 1C).

### Microarray data

We previously developed a microarray analysis of control and salt-adapted tobacco BY-2 cell cultures (Garcia de la Garma et al., 2015). From those data, we have re-analyzed the differential gene expression of AGPs and fasciclin-like proteins (Table 1). We have identified 39 probes as putative AGPs and fasciclin-like proteins. Strikingly, 45% of them were down-regulated in salt-adapted cells and none was up-regulated. Among them, only two classical AGPs were down-regulated in salt-adapted cells (AGP20 and AGP22-like). However, the majority of the down-regulated probes were putative fasciclin-like proteins. Two fasciclin-like probes, C9778_s_at and C5409_at, showed a high homology with the gene AtFLA1/SOS5 involved in proper cell expansion under salt stressed conditions. The differential expression of non-regulated probes is showed in Supplementary Table 1. We have also analyzed the differential gene expression of phospholipase C (Table 1). We identified two different probes as putative phospholipases C that were up-regulated in salt-adapted cells.

**Table 1:** Differential expression of AGP and phospholipase C genes in salt adapted cells in comparison with control cells growing in the log phase.

**Id. array**	**Ref *A. thaliana***	**Ref. SGN**	**Gene**	***p*-value**	**Fold change**
C9580_s_at	AT5G53250	SGN-U430703	arabinogalactan peptide 22-like	7.63E-08	−72.8751
C3546_s_at	AT4G12730	SGN-U451793	fasciclin-like arabinogalactan-protein 2 (Fla2)	5.52E-07	−8.0373
EB442676_at	AT4G12730	SGN-U451794	fasciclin-like arabinogalactan-protein 2 (Fla2)	1.56E-07	−6.60979
EB428861_s_at	AT4G12730	SGN-U451793	fasciclin-like arabinogalactan-protein 2 (Fla2)	0.00450591	−4.7504
BP532691_at	AT4G12730	SGN-U433024	fasciclin-like arabinogalactan-protein 1 (Fla1)	4.24E-05	−2.3124
C3697_s_at	AT2G04780	SGN-U443268	fasciclin-like arabinogalactan-protein 7 (Fla7)	2.97E-05	−2.19974
C5736_s_at	AT5G06390	SGN-U444640	FLA17 (fasciclin-like arabinogalactan-protein 17 precursor)	1.84E-06	−2.81563
EB435183_x_at	AT5G06390	SGN-U444640	FLA17 (fasciclin-like arabinogalactan-protein 17 precursor)	1.28E-05	−2.41498
C5049_at	AT5G06390	SGN-U444912	FLA17 (fasciclin-like arabinogalactan-protein 17 precursor)	0.000162973	−2.01721
C9778_s_at	AT5G55730	SGN-U433090	fasciclin-like arabinogalactan-protein 1 (Fla1)	8.13E-06	−2.25681
C528_s_at	AT5G55730	SGN-U433023	fasciclin-like arabinogalactan-protein 1 (Fla1)	5.18E-05	−2.1092
C5409_at	AT5G55730	SGN-U433089	fasciclin-like arabinogalactan-protein 1 (Fla1)	1.96E-06	−3.16571
C6841_at	AT3G61640	SGN-U450698	AGP20 (ARABINOGALACTAN PROTEIN 20)	3.89E-06	−2.80927
C10056_s_at	AT1G03870	SGN-U428649	fasciclin-like arabinogalactan-protein 9 (Fla9)	5.93E-08	−1.7524
C10056_at	AT1G03870	SGN-U428649	fasciclin-like arabinogalactan-protein 9 (Fla9)	1.94E-06	−1.78336
BP532691_s_at	AT4G12730	SGN-U433024	fasciclin-like arabinogalactan-protein 1 (Fla1)	0.000398575	−1.92305
C3697_at	AT2G04780	SGN-U443268	fasciclin-like arabinogalactan-protein 7 (Fla7)	3.41E-05	−1.94324
TT22_D05_at	AT4G36945	SGN-U451624	phospholipase C	1.94E-05	8.39226
AF223351_at	AT3G08510	SGN-U431680	ATPLC2 (PHOSPHOLIPASE C2)	2.05E-07	4.61524

*All data were analyzed using Benjamini and Hochberg (1995) test, FDR > 0.01*.

### Subcellular location of AGPs

**Figure 2:**
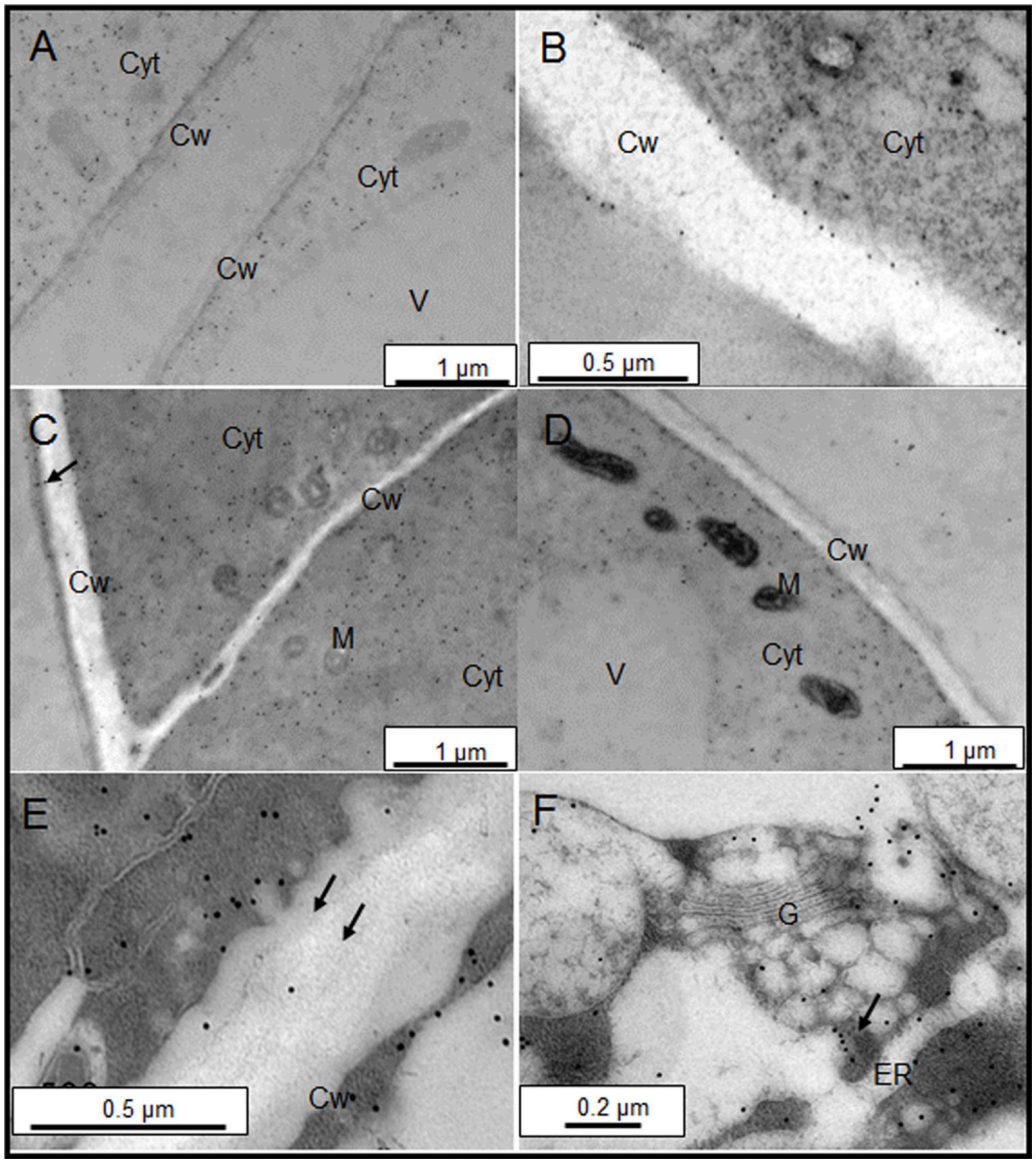
Immunogold labeling of JIM13 AGP epitope on samples of control **(A,B)** and salt-adapted **(C–F)** tobacco BY-2cells. **(A)** Control cells showing abundant labeling in the cytoplasm. **(B)** Detail of cell wall showing a high labeling at the plasma membrane and tonoplast. **(C)** Salt-adapted cells showing labeling in the cytoplasm and the extracellular matrix (arrow). **(D)** Salt-adapted cells showing labeling in the cytoplasm. **(E)** Detail of vesicles showing a high labeling close to the plasma membrane (arrows). **(F)** High labeling of JIM13 epitope in the endomembrane system. Cyt, Cytoplasm; Cw, Cell Wall; G, Golgi, ER, endoplasmic reticulum; M, Mitochondria; V, Vacuole.

**Figure 3:**
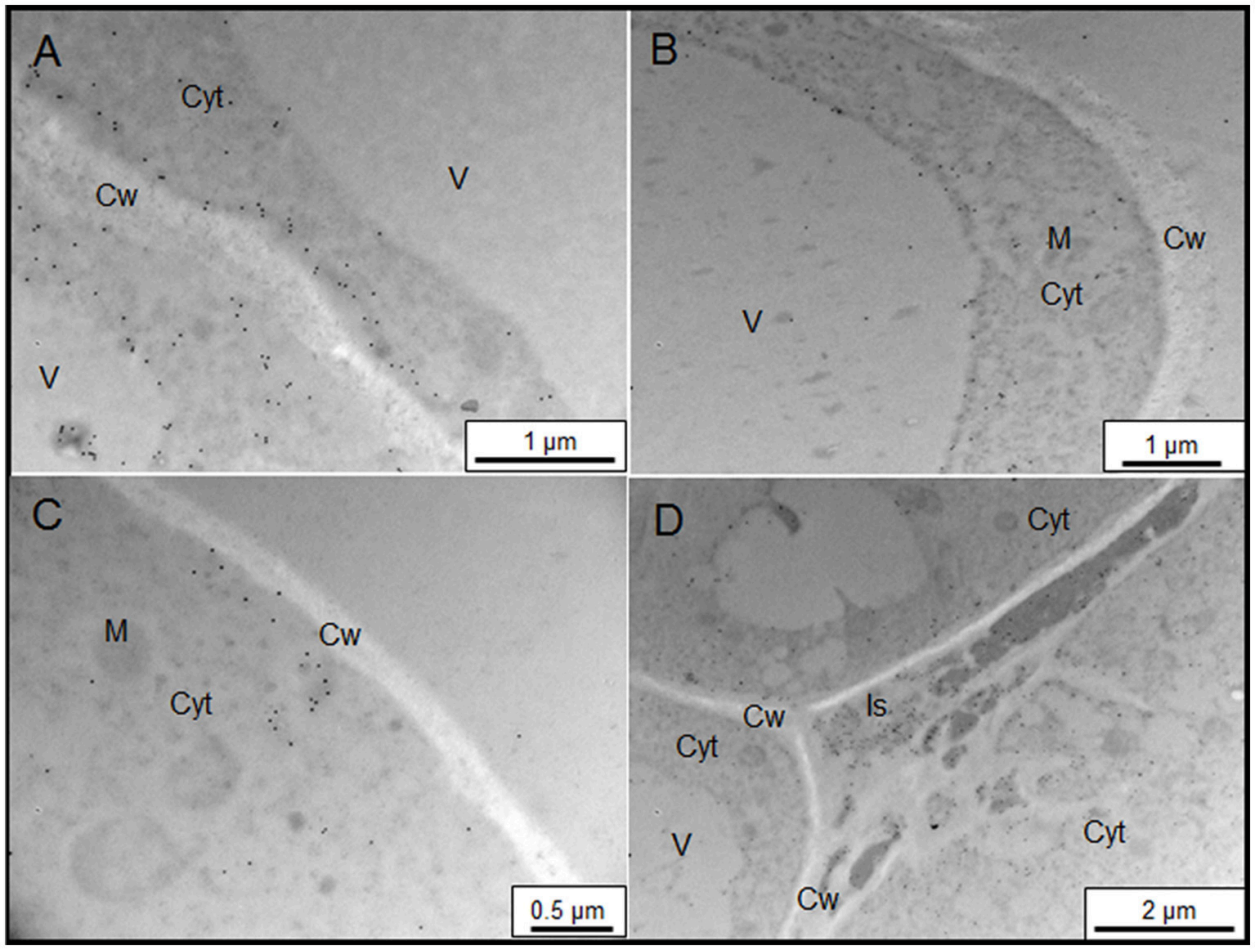
Immunogold labeling of JIM15 AGP epitope on samples of control **(A,B)** and salt-adapted **(C,D)** tobacco BY-2 cells. **(A)** Detail of cell wall showing a high labeling at the plasma membrane. **(B)** General view of control cells showing abundant labeling in the cytoplasm. **(C)** Details of plasma membrane with low labeling but showing abundant vesicles with high labeling. **(D)** JIM15 epitope secreted to the apoplast in three-way cell-cell junction. Cyt, Cytoplasm; Cw, Cell Wall; Is, Intercellular space; M, Mitochondria; V, Vacuole.

The sub-cellular distribution of two epitopes, JIM13, and JIM15, were analyzed by immunogold labeling (Figures 2, 3). JIM13 showed the higher density of labeling, but both antibodies showed a similar distribution. AGPs were abundant in the cytoplasm, showing a higher density in control cells in comparison with salt-adapted cells (Figures 2A,D, Table 2). Tonoplast labeling was also higher in control cells than salt-adapted cells (Table 2). A higher magnification analysis showed that JIM13 epitope was abundant in the plasma membrane of control cells and that salt-adapted cells showed lower labeling than control cells (Figures 2B,C, Table 2). Furthermore, salt-adapted cells showed abundant vesicles close to the plasma membrane labeled JIM13 and a well-developed endomembrane system with an important labeling of JIM13 in the trans-golgi region (Figures 2E,F). AGPs were also abundant in the intercellular spaces of salt-adapted cells (Figure 3D).

**Table 2:** Quantitative analyses of immunogold labeling distribution of arabinogalactan proteins (JIM13 and JIM15).

**Number of gold particles**			
	**Cytoplasm (μm^2^)**	**Plasma membrane (μm)**	**Tonoplast (μm)**
**JIM13**			
Control	15.14 ± 0.63a	3.64 ± 0.41a	4.37 ± 0.40a
Salt-Adapted	12.06 ± 0.87b	2.67 ± 0.19b	2.43 ± 0.14b
**JIM15**			
Control	6.06 ± 0.44a	2.63 ± 0.24a	2.41 ± 0.16a
Salt-Adapted	3.33 ± 1.46b	1.37 ± 0.07b	1.39 ± 0.06b

*Mean values ± SD. (n = 10) of control and salt-adapted cells are shown. Means without a common letter are significantly different by Tukey test (p < 0.05)*.

### Regulation of AGPs excretion by phospholipase C

**Figure 4:**
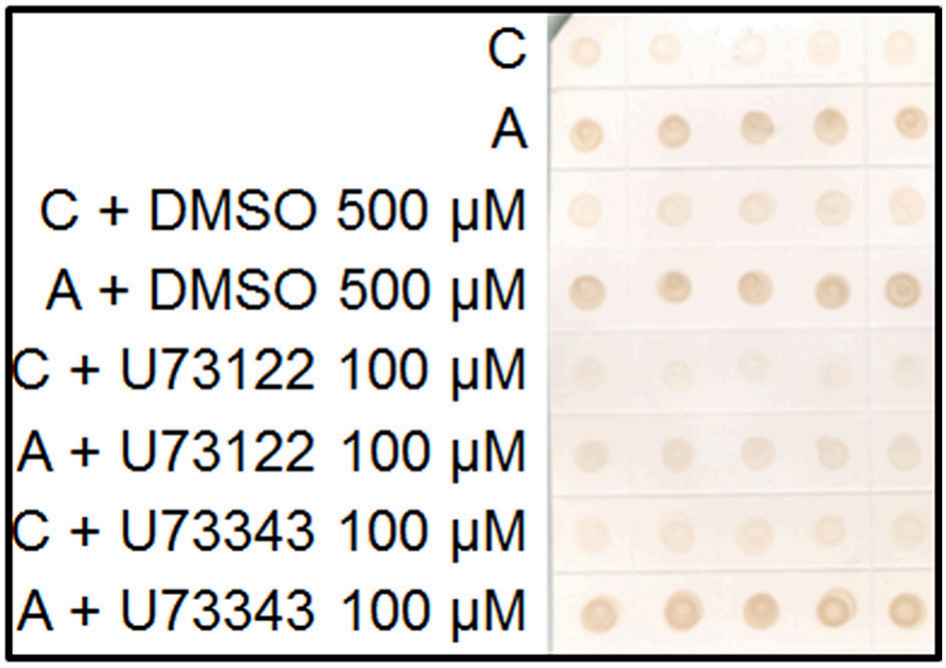
Immuno-dot blot analysis with JIM13 on samples of control (C) and salt-adapted tobacco BY-2 cells (A) treated with the inhibitor of phospholipase C, U73122 and its inactive analog, U73343. Five samples per each treatment were collected from culture medium of cells growing during 24 h.

Plasma membrane AGPs are anchored by a GPI molecule to the plasma membrane and can be liberated to the apoplast by the action of the phospholipases. We used a specific inhibitor of the phospholipase C, U73122 (Ghars et al., 2012). Salt-adapted cells showed a drastic reduction of AGP excretion to the culture medium. However, the treatment with U73343, the inactive analog of U73122, showed a similar pattern to the control treatment (Figure 4).

### Effect of Yariv treatments in control and salt-adapted cells

**Figure 5:**
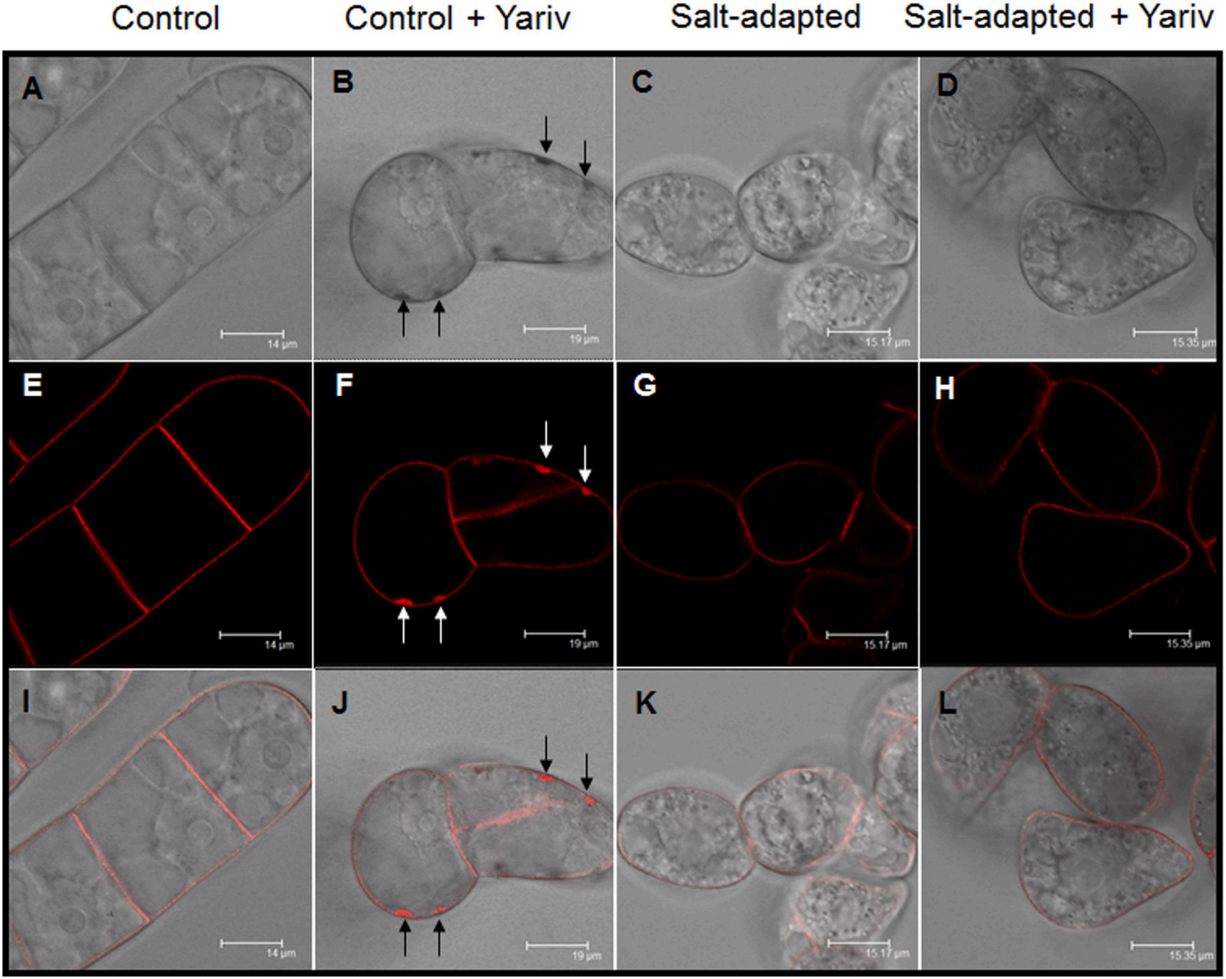
Cellular localization by fluorescence microscopy of Yariv precipitates on samples of control **(A,B,E,F,I,J)** and salt-adapted **(C,D,G,H,K,L)** tobacco BY-2 cells treated and non-treated with 100 μM Yariv reagent for 24 h. **(A**–**D)** DIC images; **(E–H)** FM4-64 fluorescence images; **(I–L)** Overlapping of DIC and fluorescence images. Arrows in **(B,F,J)** indicate the location of the precipitates of Yariv reagent.

**Figure 6:**
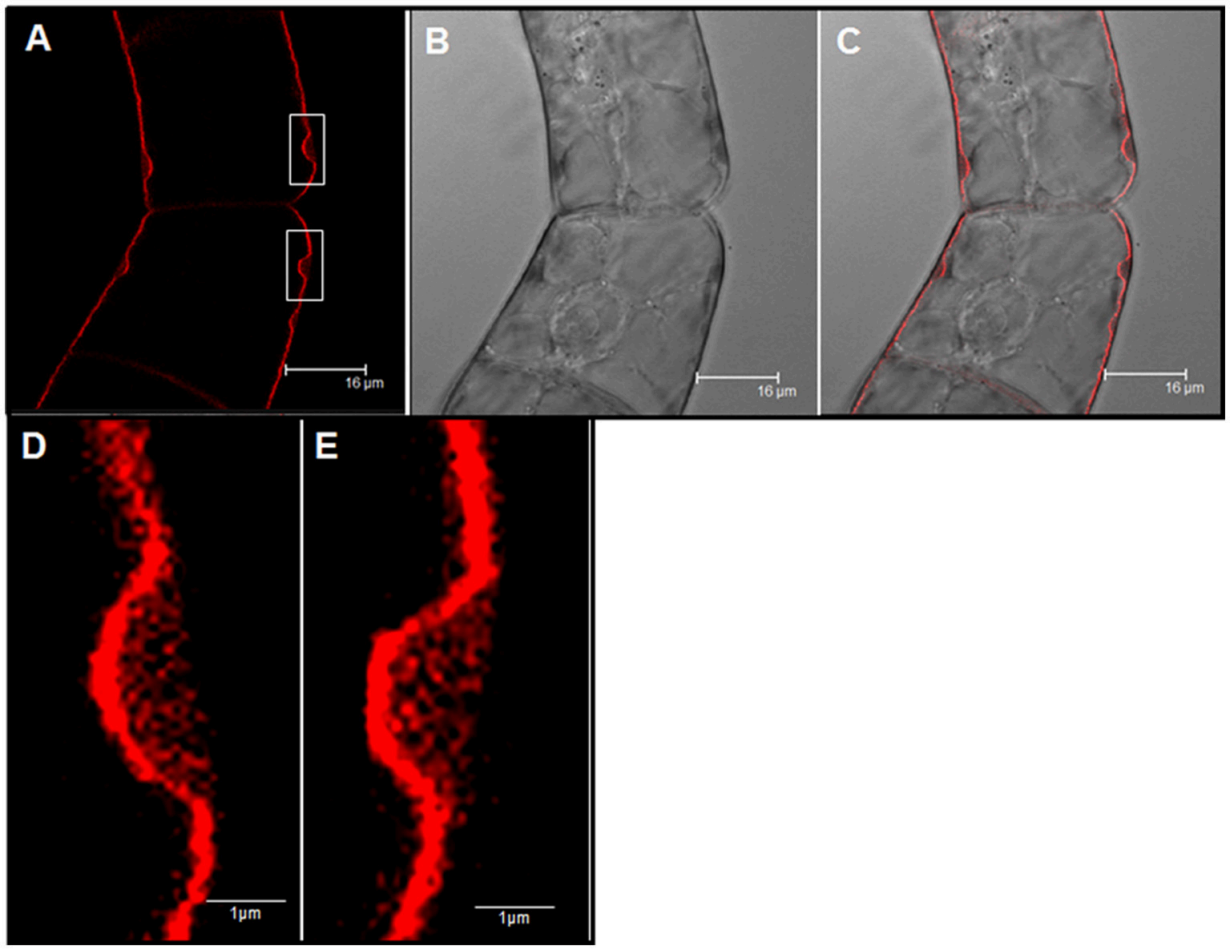
Cellular localization by fluorescence microscopy of Yariv precipitates on samples of control tobacco BY-2 cells treated with 100 μM Yariv reagent for 24 h. **(A)** FM4-64 fluorescence images (Yariv precipitates, white squares). **(B)** DIC image. **(C)** Overlapping of DIC and fluorescence images. **(D,E)** A high magnification from image **(A)** of Yariv precipitates showing a coil structure.

We analyzed the effect of Yariv reagent in the control and salt-adapted cell cultures. After 24 h of the Yariv treatment, cell viability was significantly reduced in control cells but no effect was observed in salt-adapted cells (Table 3). Microscopical analysis of these cells showed that a brown staining was observed in the periphery of the control cells (Supplementary Figures 1a,c) that seems to be located at the plasma membrane. However, salt-adapted cells did not show any alteration or staining (Supplementary Figures 1b,d). To confirm the localization of the brown precipitate, we used the fluorochrome FM4-64, which specifically labels the plasma membrane. In control cells treated with the Yariv reagent, FM4-64 fluorescence overlapped with the brown precipitates (Figure 5). A higher magnification of this area showed that FM4-64 labeling was distributed as a coil structure, suggesting the presence of membranes in the brown precipitates (Figure 6).

**Table 3:** Relative percentage of living cells of tobacco cells treated for 24 h. with 100 μM Yariv reagent.

	**Control**	**Control+ β-D-glucosyl Yariv**	**Salt-adapted**	**Salt-adapted+ β-D-glucosyl Yariv**
**Relative %**	100a	87.2b	100a	100a

*Mean values. (n = 3) of control and cell treated with Yariv reagent are shown. Means without a common letter are significantly different by Tukey test (p < 0.05)*.

**Figure 7:**
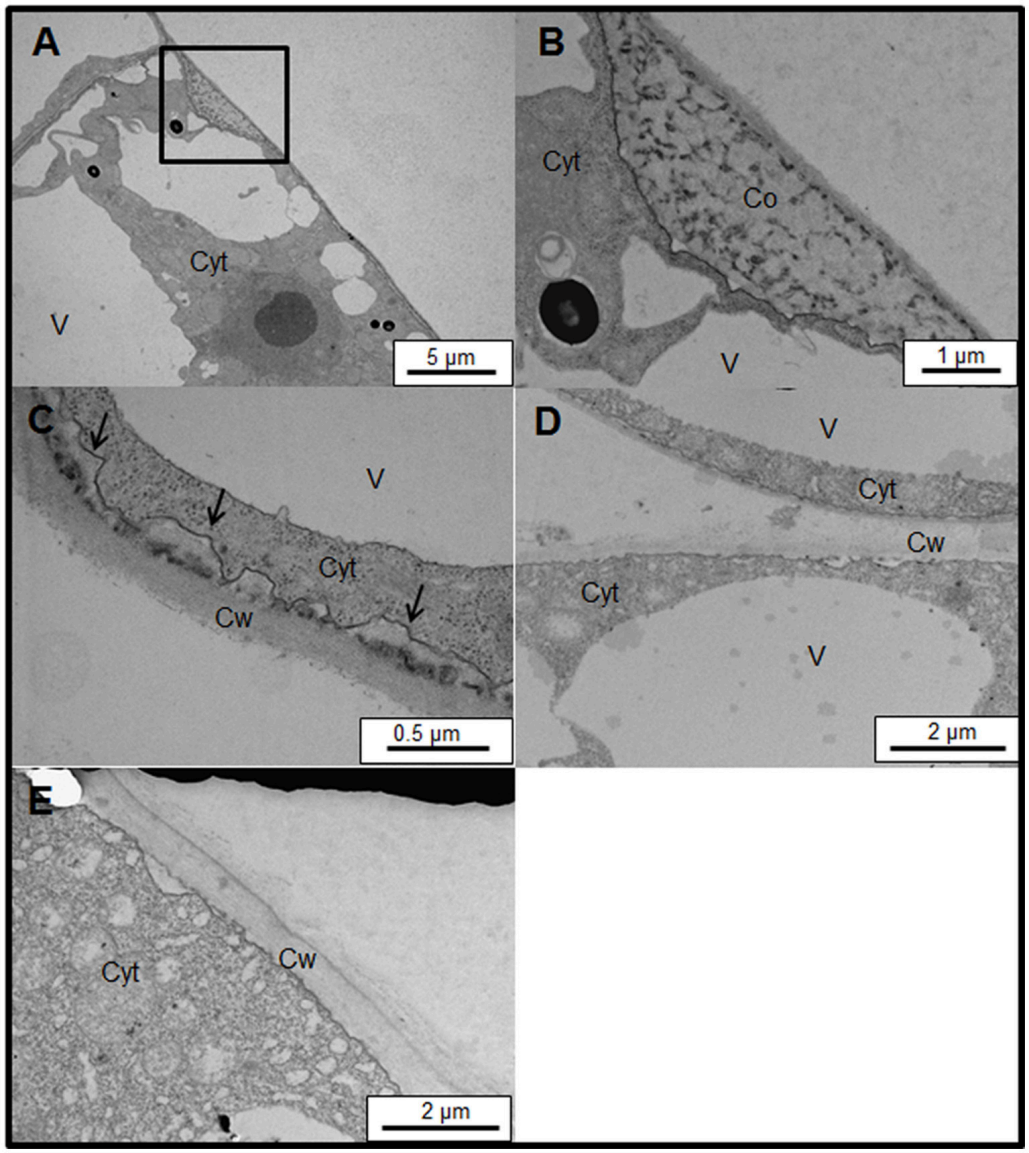
Subcellular location of Yariv reagent precipitation by transmission electron microscopy on samples of control **(A–C)** and salt-adapted **(D,E)** tobacco BY-2 cells. **(A)** Ultrastructure of control tobacco cells treated with 100 μM Yariv reagent for 24 h. Yariv precipitate is located between the plasma membrane and cell wall (see square). **(B)** Detail from image **(A)** showing the coil structure of Yariv precipitate showing a coil structure formed by dense filaments. **(C)** A high magnification showing the detachment of plasma membrane from the cell walls (arrows) in control tobacco cells treated with 100 μM Yariv reagent for 24 h. **(D,E)** Ultrastructure of salt-adapted tobacco cells treated with 100 μM Yariv reagent for 24 h. Yariv reagent precipitates were not observed at the plasma membrane. Co, Coil structure of Yariv precipitates; Cyt, Cytoplasm; Cw, Cell Wall; V, Vacuoles.

To analyze the structure of the brown precipitates we studied the ultrastructure of control and salt-adapted cells. The ultrastructural analysis of control cells confirmed the coil structure of the brown precipitates. The coil structure was located between the plasma membrane and cell walls (Figures 7A,B). A dense stained filament is observed in the coil structure. These structures resemble the images observed with laser confocal microscopy (see Figure 6). A higher magnification showed a small region with a dense labeling between the plasma membrane and cell walls, inducing the detachment of plasma membrane from the cell walls (Figure 7C). These structures might be the beginning of the formation of the coil structures in control cells treated with the Yariv reagent. Unlike control cells, the ultrastructural analysis of salt-adapted cells confirmed the absence of the coil structures between the plasma membrane and cell walls (Figures 7D,E).

**Figure 8:**
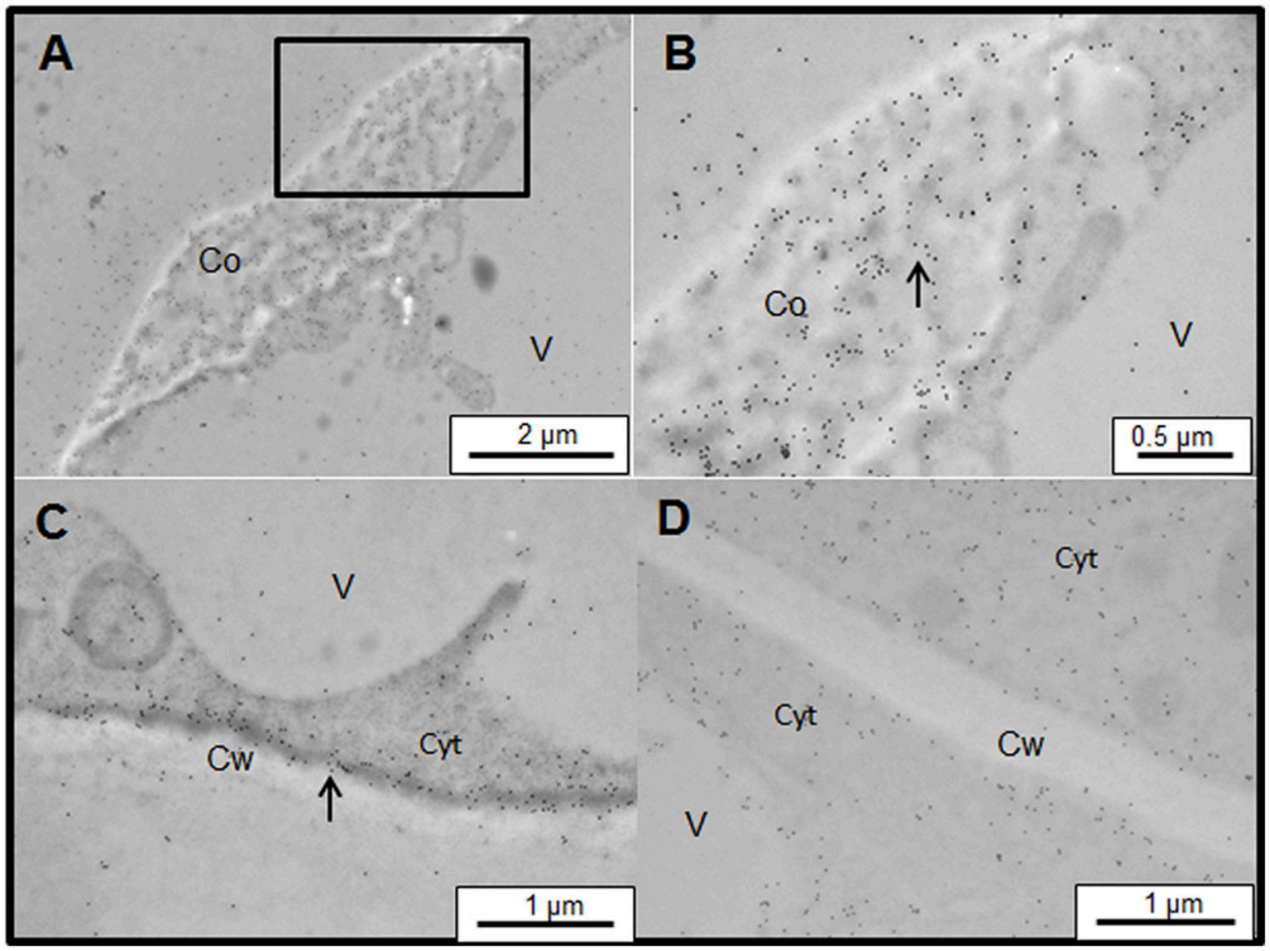
Immunogold labeling of JIM13 AGP epitope associated with Yariv precipitates on samples of control **(A–C)** and salt-adapted **(D)** tobacco BY-2 cells. **(A)** JIM13 epitope was abundantly located on Yariv precipitates of control tobacco cells treated with 100 μM Yariv reagent for 24 h. **(B)** Detail from image **(A)** showing that JIM13 labeling was specifically located in the dense filament (arrow). **(C)** JIM13 labeling was abundantly located between the plasma membrane and cell wall (arrow). **(D)** salt-adapted tobacco cells treated with 100 μM Yariv reagent for 24 h showed a similar distribution of JIM13 epitope to untreated cells (see Figure 2).

Finally, the presence of AGPs in the coil structures was studied by immunogold labeling with JIM13. The immunolabeling was strongly detected in the coil structures (Figure 8A), a higher magnification of the coil structures showed that JIM13 labeling was specifically located in the dense filament previously observed in the ultrastructural study (Figures 8B). Similarly, the dense structures observed between the plasma membrane and cell walls showed a strong labeling with JIM13 (Figure 8C). However, salt-adapted cells showed a similar pattern of distribution of JIM13 to the cells not treated with Yariv (Figure 8D).

## Discussion

In the present study, we suggest different mechanisms of action of AGPs during salt-adaptation. In the following sections, we describe the possible mechanism of AGP accumulation in the culture medium and its role, the possible action of AGPs as sodium carrier in the cytoplasm through endocytic vesicle trafficking, and we also analyze the differential response between control and salt-adapted cells to Yariv reagent treatments.

### Salt-adaptation induces a progressive accumulation of AGPs in the culture medium through the action of phospholipase C, and these may be acting as pectin plasticizers

Different studies in the literature have focused on the accumulation of AGPs in culture medium during salt-adaptation in cell cultures (Zhu et al., 1993; Lamport et al., 2006; Kimura et al., 2008; Zargochev and Odjakova, 2011). However, some important discrepancies are produced between them and our study regarding the accumulation of AGPs in the culture medium. These discrepancies are mainly due to different ways of evaluating the total amount of AGPs in the culture medium. Therefore, we consider that a correct quantification of AGPs in the culture medium should be made. In our opinion, AGPs concentration must be quantified per unit of biomass and cellular size, and cytoplasm/vacuoles ratio should be also taken into consideration.

Our major discrepancy with the literature is the level of AGPs accumulation in the culture medium observed by Zhu et al. (1993) in salt-adapted tobacco cell cv Wisconsi. These authors reported a high reduction in plasma membrane-bound AGPs and the decrease of AGPs accumulation in the culture medium. Our data are in agreement with the reduction in plasma membrane-bound AGPs. However, we observed a high accumulation of AGPs in the culture medium of salt-adapted cells. The discrepancy can be explained as Lamport et al. (2006) proposed, but Zhu et al. (1993) did not make the comparison based on AGPs yields per unit of biomass.

On the other hand, cellular adaptation to high saline concentrations implies a significant cell volume reduction. In our previous study, we observed that salt-adapted cells showed a reduced size and an altered ultrastructure (Garcia de la Garma et al., 2015). Cellular volume of salt-adapted cell is about 1/2 to 1/3 that of control cells, depending on the growing phase. Similarly, Binzel et al. (1985) reported that *N. tabacum* cv Wisconsi adapted to 428 mM of NaCl showed a much lower cellular size in comparison with control cells. Moreover, salt-adapted BY-2 cells showed a much higher cytoplasm/vacuole ratio (about 2.5-fold). However, Lamport et al. (2006) used the same fresh weight of control and salt-adapted cells, assuming that control and salt-adapted cells have the same cell size. In any case, the concentration of AGPs in the culture medium of tobacco BY-2 salt-adapted cells is significantly higher than in control cells.

So, how are AGPs accumulated in the culture medium of salt-adapted cells? We propose that AGPs are accumulated in the culture medium through an exocytic pathway and released into the culture medium by the cleavage of AGPs from the plasma membrane mediated by phospholipase C. Prior to AGPs excretion by exocytosis and plasma membrane cleavage, AGPs are synthetized in the RER and modified by the Golgi apparatus and then transported to the plasma membrane by vesicles. We are able to draw up a hypothesis of a dynamic flow of AGPs from their synthesis in the endomembrane system to the apoplast, based on our results and the previous literature. Garcia de la Garma et al. (2015) showed that BY-2 salt-adapted cells have a highly abundant endomembrane system, with a well-developed Golgi apparatus and endoplasmic reticulum. AGPs are characterized by the O-glycosylation of the protein backbone which takes place post-translationally in the Golgi apparatus. Our immunogold labeling using the monoclonal antibodies JIM13, JIM15, and LM2 demonstrates that these epitopes are present in the Golgi membranes, Golgi derived-vesicles, and ER. Similarly, Samaj et al. (2000) using immunogold labeling with the monoclonal antibodies JIM13 and LM2 in maize and *Drosera capensis*, locate these epitopes in the ER and Golgi membranes and derived-vesicles, so some AGPs may be transported as a component of the endomembrane flow participating in the AGP exocytosis. Interestingly, Winicur et al. (1998) observed that BY-2 cells deprived of 2,4-D showed a higher labeling with JIM13 of Golgi and Golgi derived vesicles and a higher accumulation of AGPs in the culture medium. AGPs may be anchored to the plasma membrane by a glycosylphosphatidylinositol (GPI) and the GPI-AGPs may be susceptible to cleavage by endogenous phospholipase C, releasing the previously membrane-bound protein into the extracellular medium (Sherrier et al., 1999; Darjania et al., 2002). On the other hand, our results show that phospholipase C inhibitions drastically reduced the AGPs accumulation in the culture medium. Moreover, microarrays data confirm that phospholipase C genes were up-regulated in salt-adapted cells probably showing a higher phospholipase C activity. Altogether, immunogold labeling and biochemical data suggest that salt-adapted cells have strongly induced the exocytic pathway and AGPs linked to the plasma membrane can be removed by the action of phospholipase C, inducing a higher accumulation of AGPs in the extracellular medium.

Little is known about the possible role of the excreted AGPs in the apoplast. The most plausible hypothesis was proposed by Lamport et al. (2006), that of pectin plasticizers. Various studies are in agreement with this hypothesis. One of the most relevant was that with the fasciclin-like AGP SOS5 (Salt-Overly Sensitive 5) mutant, which was isolated in a screening for Arabidopsis salt-hypersensitive mutants (Shi et al., 2003). This protein was located on the outer surface of the plasma membrane and is required for normal cell expansion. Salt-treated SOS5 mutants showed that the root tips swelled and root growth was arrested. It has been suggested that SOS5 mediates adherence through its interaction with the cell wall pectins (Griffiths et al., 2014). Therefore, the interaction between pectins and AGPs seem to be critical for the regulation of cell expansion. McCann et al. (1994) observed that salt-adapted tobacco cells showed important modifications in pectin esterification in the cell walls. It was suggested that AGPs may be reducing the formation of pectate gels and, therefore, affecting cell wall extension (Serpe and Nothnagel, 1994). We observed that AGPs are highly accumulated in the apoplast of salt-adapted cells mainly during the exponential and stationary phases. Strikingly, cell expansion is mainly produced during these phases. Therefore, we suggest that AGPs accumulation might be regulating the cell wall extensibility, so affecting the pectin network as plasticizers and facilitating the cell expansion under a high osmotic medium such as 278 mM of NaCl.

However, other possible roles of AGPs in the apoplast should not be discarded. For example, GPI-anchored AGPs could also participate in signaling pathways in plants. It has been observed that purified AGPs from embryogenic cell can stimulate non-embryogenic cells to undergo embryogenesis (Mallon et al., 2013; Shu et al., 2014).

### AGPs are actively moving from the plasma membrane to the tonoplast. Are AGPs acting as sodium carrier from the apoplast to the vacuoles?

In the previous section, an exocytic mechanism seems to be critical for AGPs excretion to the apoplast. Parallel to the exocytosis, endocytic mechanism of AGPs have also been suggested for BY-2 cells (Lamport and Varnai, 2013). This hypothesis agrees with previous studies that showed evidence that AGPs may also be moving from the plasma membrane to the tonoplast.

One of these studies was Herman and Lamb (1992). These authors using a monoclonal antibody named 16.4B4, which labels a glycan epitope of a *N. tabacum* 50kD AGP plasma membrane protein demonstrated, through immunogold labeling, that this epitope was located at the plasma membrane surface (Herman and Lamb, 1992). This antigen was also located in multivesicular bodies emerging from the plasma membrane. Moreover, the multivesicular bodies were also observed connecting with the tonoplast and inside of the vacuoles. Similarly, Samaj et al. (2000) located the JIM13 and LM2 epitopes in vesicles, multivesicular bodies and tonoplasts. These authors proposed that some AGPs are transported from the plasma membrane to the tonoplast. A similar flow of AGPs was recently proposed by Lamport and Varnai (2013) in tobacco BY-2 cells. Strikingly, these authors have observed that AGPs contain about 30 Ca^2+^ atoms linked per each AGP molecule. However, when cells are grown in a saline medium, Na^+^ is able to replace Ca^2+^ in the AGP molecules (Lamport et al., 2014). These authors demonstrated that 75 mM of NaCl removes approximately 50% of calcium from a carrot AGP-Ca^2+^.

**Figure 9:**
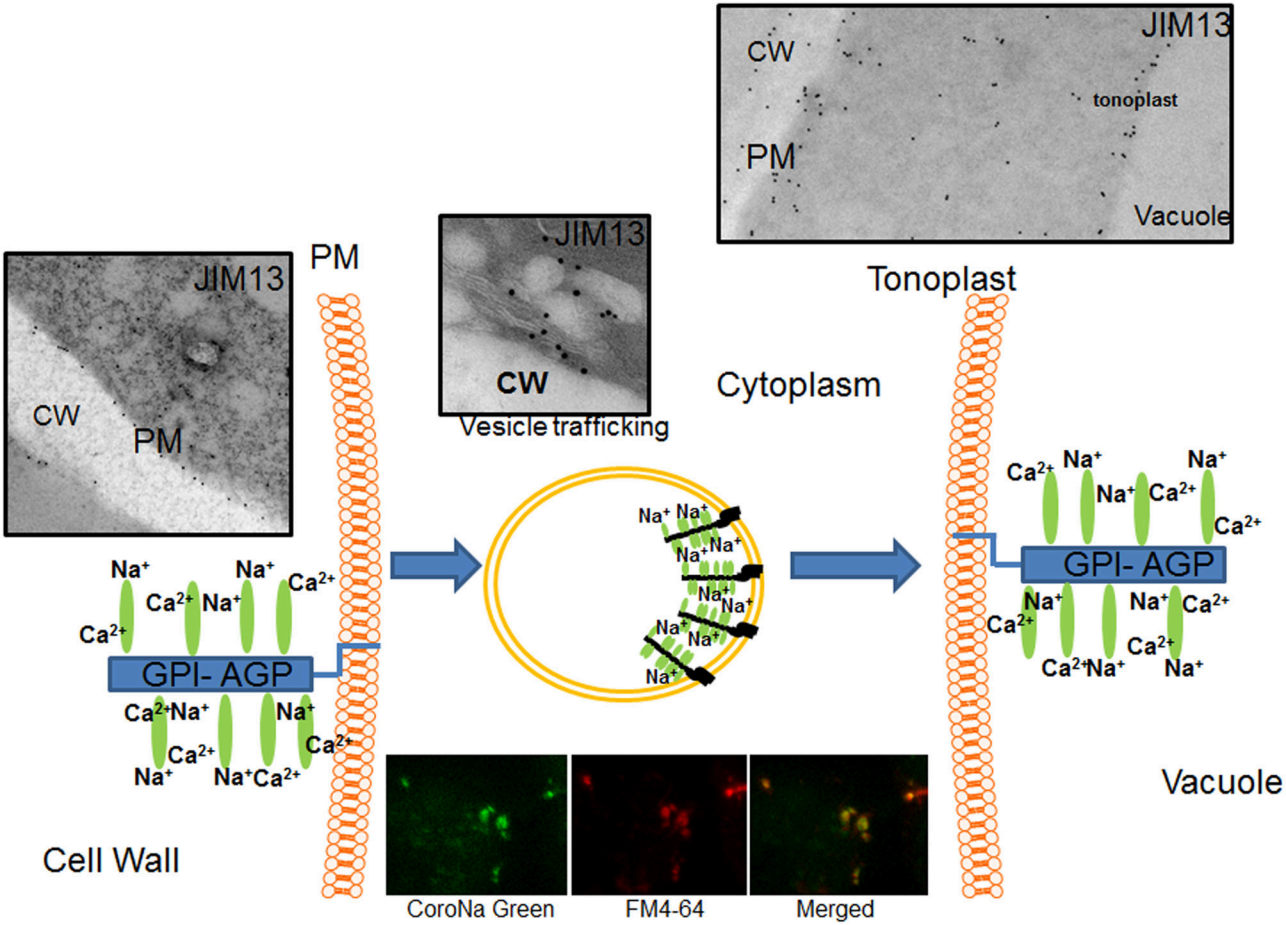
Proposed model of AGPs as sodium carrier from the apoplast to the vacuoles in salt-adapted tobacco cells. AGPs bind calcium at the plasma membrane but it was partially removed by high sodium concentration in the apoplast. Plasma membrane linked AGPs are internalized by endocytosis transporting sodium by vesicle trafficking to the tonoplast and then liberating sodium in the vacuole. Sodium was located using the fluorochrome Asante Natrium-Green and vesicles were labeled using FM4-64. Cw, Cell Wall; Pm, Plasma membrane.

Recently, Bassil et al. (2012) proposed that endosomal vesicles that contain NHX isoforms, involved in Na^+^/H^+^ antiport, could be sequestering the excess cytosolic Na^+^ within the endosomal vesicles that subsequently fuse to the vacuole, thus contributing to reducing high cytosolic Na^+^ concentrations. Our data of AGPs location and distribution are in agreement with the hypothesis of AGPs transport from the plasma membrane to the tonoplast, as the distribution of AGPs in vesicles and the presence of AGPs in the tonoplast. We previously observed that sodium is highly accumulated in the vacuoles and vesicles of salt-adapted cells (Garcia de la Garma et al., 2015). In the present study, we confirmed with a new fluorochrome (Asante Natrium-Green) the *in vivo* formation of small vesicles containing sodium (Garcia de la Garma et al., 2015; see inset in Figure 9). Therefore, we suggested that Na^+^ might be transported from the apoplast to the vacuoles following an endocytic pathway (see Figure 9). In this model, sodium is linked by GPI-AGPs anchored to the external face of the plasma membrane removing calcium molecules from the glycan moiety of the AGPs (left, in Figure 9). Then, GPI-AGPs-Na^+^ complex is internalized by endocytosis and originating vesicles enriched in sodium (middle, in Figure 9). Finally, these vesicles move throughout the cytoplasm (following the endocytic pathway) and they merge with the tonoplast (right, in Figure 9), liberating the sodium to the vacuoles. New studies will be necessary in the future to confirm the co-location of AGPs and sodium inside the endocytic vesicles. Currently, we are unable to quantify the possible contribution of this mechanism to the salt-adaptation mechanism but we consider it may be significantly contribute to the sodium homeostasis mechanism.

### AGP gene expression is down-regulated in salt-adapted cells and could be regulated by ABA

We have developed a transcriptome study of AGPs to analyze if AGPs production is differently regulated in salt-adapted cells. The tobacco microarray used in our study has 39 probes related as AGPs and Fasciclin-like proteins (Table 1). Our data demonstrate that 17 probes were down-regulated and none was up-regulated. Therefore, these results indicate that AGP expression is down-regulated by salt stress. These data are in agreement with the significantly lower concentration of the epitopes JIM13 and JIM15 located in the cytoplasm of salt-adapted cells. However, AGPs were accumulated in the culture medium of salt-adapted cells. In our opinion, the higher accumulation of AGPs in the culture medium during the exponential phase could be inducing a negative feedback, so reducing the AGP gene expression observed in the microarray data. The over-accumulation of AGPs in the culture medium during the exponential phase could be negatively inducing an excessive expansion of cell walls under the high osmotic conditions. The cell wall expansion can be inducing a much higher water uptake increasing the cell volume. Therefore, sodium uptake and/or a higher accumulation of osmolytes should be also increased to equilibrate the osmotic effect of culture medium. Different studies have developed a molecular analysis of the AGPs gene expression during salt treatments, showing down and/or up-regulation of AGPs. Our results are in agreement with Ouyang et al. (2007) when following an SSH and microarray approaches. These authors identified 5 different AGPs that were strongly repressed in salt treated tomato roots. Similarly, Huang et al. analyzed the differential expression of 19 putative fasciclin-like proteins in *Gossypium hirsutum* in plants treated with different phytohormones and NaCl (Huang et al., 2008). These authors observed that several fasciclin-like proteins were down regulated by NaCl and ABA. In *Arabidopsis thaliana*, different studies have also observed that AtFLA1/2/8 transcript levels were down-regulated in the presence of ABA and AtFLA2/8/13/20 transcript levels were down-regulated by salt treatments (Johnson et al., 2003, Xin et al., 2005, Ma and Zhao, 2010). Similarly, two rice OsFLA (OsFLA10/18) and two wheat TaFLA3/4 genes under salinity were significantly down-regulated (Faik et al., 2006; Ma and Zhao, 2010). Meanwhile, the expression levels of five FLA genes in *Populus trichocarpa* roots (PtrFLC2/12/20/30) were up-regulated under salt stress (Zang et al., 2015). Therefore, it seems that AGPs gene expression is in general repressed by salt treatments in several species but not in others. The mechanism of regulation of AGPs gene expression remains unknown. However, some of these studies have suggested that AGPs are involved in ABA signal transduction pathways (Xin et al., 2005; Huang et al., 2008; Ma and Zhao, 2010). ABA is an important hormone that regulates many downstream genes, and is involved in response to abiotic stresses throughout the plant kingdom. It is well-known that many cellular responses are modulated by the ABA signal transduction pathway during salt-adaptation and salt tolerance (Danquah et al., 2014). Interestingly, we have previously observed that BY-2 salt-adapted cells showed a high accumulation of ABA, showing that the phospholipases pathway was activated (Garcia de la Garma et al., 2015). Therefore, we suggest that ABA may be involved in the AGPs down-regulation observed in salt-adapted cells.

### Yariv reagent induces cell death through AGPs aggregation in control cells but not in salt-adapted cells

Maurer et al. (2010) have observed that Brazilian pine suspension culture cells treated with Yariv reagent induced cell death. In a previous study, Gao and Showalter (1999) showed that Yariv reagent treatment also induced cell death in Arabidopsis cell cultures. These authors observed by electron microscopy that some cells showed a cytoplasm detachment. Similarly, we have observed in the control BY-2 that cells treated with 100 μM of Yariv reagent showed some cells with a similar cytoplasm detachment. However, we have observed that at a different point of the cell surface, Yariv reagent is accumulated, forming coils of plasma membrane and Yariv reagent. Some authors have proposed that Yariv reagent is aggregating the AGPs and disturbing the scaffold proteins and altering the alignment of cortical microtubules and cellulose microfibrils (Sardar et al., 2006, Nguema-Ona et al., 2007, Driouich and Baskin, 2008). Our study presents the first ultrastructural and biochemical evidence of this mechanism of action of the Yariv reagent. Yu and Zhao (2012) observed that tobacco zygote and proembryo cells treated with Yariv altered cell plate formation during cell division, probably due to modifications in the endosomes distribution during cell plate formation. We propose that the Yariv reagent is inducing cell detachments and facilitating plasma membrane AGPs aggregations, forming a complex coil of membranes that finally alters the plasma membrane structure and induces cell death.

Strikingly, salt-adapted cells were unaltered by Yariv reagent treatments. We mentioned above that salt-adapted cells showed a much lower content of AGPs at the plasma membrane. In our opinion, the Yariv reagent is unable to form these coils of AGPs probably due to a lower density of AGPs at the plasma membrane of salt-adapted cells in comparison with control cells. We consider that this fact reinforces the hypothesis that salt-adaptation reduces the concentration of AGPs in the plasma membrane.

## Author contributions

NF, EO, and JG performed the experiments. MG contributed to immulocation of AGPs and reviewed the manuscript. EO and NF conceived and supervised the study, wrote and critically revised the manuscript.

### Conflict of interest statement

The authors declare that the research was conducted in the absence of any commercial or financial relationships that could be construed as a potential conflict of interest.
