# Supplementary material for: Arabinogalactan Proteins Are Involved in Salt-Adaptation and Vesicle Trafficking in Tobacco by-2 Cell Cultures
Source: Front Plant Sci. 2017 Jun 20;8:1092. doi: 10.3389/fpls.2017.01092 (PMC5476920; doi:10.3389/fpls.2017.01092)
Supplement: Supplementary file 1 [file Table1.PDF]

Supplementary Table 1. Differential expression of non-regulated AGP genes in salt adapted cells in comparison with control cells growing in the log phase. All data were analyzed using Benjamini and Hochberg (1995) test, FDR >0.01.

Id. Array	Ref A. thaliana	Ref. SGN	Gene	p-value	Fold Change
C7095_at	AT5G24105	SGN-U430192	AGP41	5.59E-06	-1.4807
C7095_x_at	AT5G24105	SGN-U430192	AGP41	0.00012634	-1.4509
C4996_at	AT5G06390	SGN-U449699	FLA17 (fasciclin-like arabinogalactan-protein 17 precursor)	0.0175129	-1.3976
C3986_at	AT2G04780	SGN-U443268	fasciclin-like arabinogalactan-protein 7 (Fla7)	0.011312	-1.3353
TT04_A03_s_at	AT2G35860	SGN-U447466	FLA16 (fasciclin-like arabinogalactan-protein 16 precursor)	0.000688441	-1.2510
C1352_s_at	AT5G06390	SGN-U447466	FLA17 (fasciclin-like arabinogalactan-protein 17 precursor)	0.00176872	-1.2412
TT05_A22_at	AT3G61640	SGN-U436491	AGP20 (ARABINOGALACTAN PROTEIN 20)	0.102865	-1.2111
EB681606_at	AT2G45470	SGN-U432316	FLA8 (Arabinogalactan protein 8)	0.0339821	-1.1745
EB448541_at	AT2G45470	SGN-U432316	FLA8 (Arabinogalactan protein 8)	0.285271	-1.1461
C6637_s_at	AT3G12660	SGN-U427658	FLA14 (fasciclin-like arabinogalactan-protein 14 precursor)	0.0580468	-1.1336
C6729_at	AT2G24450	SGN-U427657	FLA3 (fasciclin-like arabinogalactan-protein 3 precursor)	0.21777	-1.1012
C63_x_at	AT5G03170	SGN-U432967	FLA11 (fasciclin-like arabinogalactan-protein 11)	0.244706	-1.0912
C3650_at	AT5G03170	SGN-U451545	FLA11 (fasciclin-like arabinogalactan-protein 11)	0.602111	-1.0734
C3670_x_at	AT5G03170	SGN-U432967	FLA11 (fasciclin-like arabinogalactan-protein 11)	0.506754	-1.0377
C6696_at	AT3G12660	SGN-U427658	FLA14 (fasciclin-like arabinogalactan-protein 14 precursor)	0.559796	-1.0369
C3906_at	AT5G03170	SGN-U449886	FLA11 (fasciclin-like arabinogalactan-protein 11)	0.867633	1.01855
EB450771_at	AT5G03170	SGN-U471095	FLA11 (fasciclin-like arabinogalactan-protein 11)	0.545581	1.02212
TT47_C01_at	AT1G03870	SGN-U428649	fasciclin-like arabinogalactan-protein 9 (Fla9)	0.563937	1.0287
C63_at	AT5G03170	SGN-U432967	FLA11 (fasciclin-like arabinogalactan-protein 11)	0.490409	1.04497
C6901_at	AT1G28290	SGN-U443601	AGP31 (Arabinogalactan-protein 31)	0.387045	1.06375
C3670_at	AT5G03170	SGN-U432967	FLA11 (fasciclin-like arabinogalactan-protein 11)	0.111565	1.07862

C6901_s_at	AT1G28290	SGN-U443601	AGP31 (Arabinogalactan-protein 31)	0,00397972	1.47147
