# Supplementary material for: Arabinogalactan Proteins Are Involved in Salt-Adaptation and Vesicle Trafficking in Tobacco by-2 Cell Cultures
Source: Front Plant Sci. 2017 Jun 20;8:1092. doi: 10.3389/fpls.2017.01092 (PMC5476920; doi:10.3389/fpls.2017.01092)
Supplement: Supplementary file 2 [file Image1.PDF]

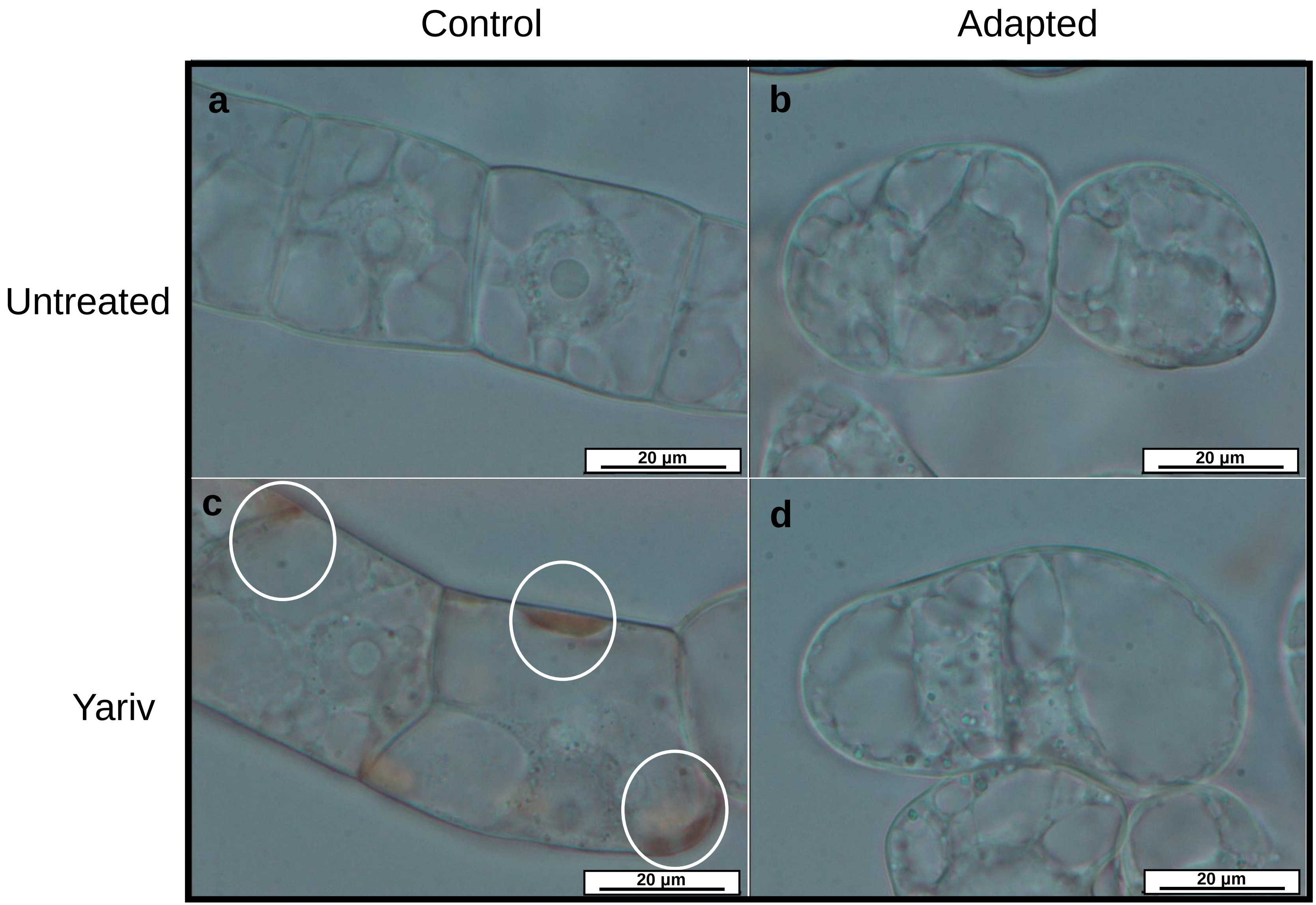

Supplementary Figure 1. Cellular localization by DIC microscopy of Yariv precipitates on samples of control (a and c) and salt-adapted (b and d) tobacco BY-2 cells. (a and b) Untreated cells. (c and d) Cells grown in the presence of 100 µM Yariv reagent for 24 hours. Circles in (c) indicate the location of the brown precipitates of Yariv reagent.
